# Validating the predictive ability of the 2MACE score for major adverse cardiovascular events in patients with atrial fibrillation: results from phase II/III of the GLORIA-AF registry

**DOI:** 10.1007/s11239-023-02866-y

**Published:** 2023-08-11

**Authors:** Wern Yew Ding, Ameenathul Mazaya Fawzy, Giulio Francesco Romiti, Marco Proietti, Daniele Pastori, Menno V. Huisman, Gregory Y. H. Lip, on behalf of the GLORIA-AF Investigators, Dzifa Wosornu Abban, Nasser Abdul, Atilio Marcelo Abud, Fran Adams, Srinivas Addala, Pedro Adragão, Walter Ageno, Rajesh Aggarwal, Sergio Agosti, Piergiuseppe Agostoni, Francisco Aguilar, Julio Aguilar Linares, Luis Aguinaga, Jameel Ahmed, Allessandro Aiello, Paul Ainsworth, Jorge Roberto Aiub, Raed Al-Dallow, Lisa Alderson, Jorge Antonio Aldrete Velasco, Dimitrios Alexopoulos, Fernando Alfonso Manterola, Pareed Aliyar, David Alonso, Fernando Augusto Alves da Costa, José Amado, Walid Amara, Mathieu Amelot, Nima Amjadi, Fabrizio Ammirati, Marianna Andrade, Nabil Andrawis, Giorgio Annoni, Gerardo Ansalone, M. Kevin Ariani, Juan Carlos Arias, Sébastien Armero, Chander Arora, Muhammad Shakil Aslam, M. Asselman, Philippe Audouin, Charles Augenbraun, S. Aydin, Ivaneta Ayryanova, Emad Aziz, Luciano Marcelo Backes, E. Badings, Ermentina Bagni, Seth H. Baker, Richard Bala, Antonio Baldi, Shigenobu Bando, Subhash Banerjee, Alan Bank, Gonzalo Barón Esquivias, Craig Barr, Maria Bartlett, Vanja Basic Kes, Giovanni Baula, Steffen Behrens, Alan Bell, Raffaella Benedetti, Juan Benezet Mazuecos, Bouziane Benhalima, Jutta Bergler-Klein, Jean-Baptiste Berneau, Percy Berrospi, Sergio Berti, Andrea Berz, Elizabeth Best, Paulo Bettencourt, Robert Betzu, Ravi Bhagwat, Luna Bhatta, Francesco Biscione, Giovanni Bisignani, Toby Black, Michael J. Bloch, Stephen Bloom, Edwin Blumberg, Mario Bo, Ellen Bøhmer, Andreas Bollmann, Maria Grazia Bongiorni, Giuseppe Boriani, D. J. Boswijk, Jochen Bott, Edo Bottacchi, Marica Bracic Kalan, Drew Bradman, Donald Brautigam, Nicolas Breton, P. J. A. M. Brouwers, Kevin Browne, Jordi Bruguera Cortada, A. Bruni, Claude Brunschwig, Hervé Buathier, Aurélie Buhl, John Bullinga, Jose Walter Cabrera, Alberto Caccavo, Shanglang Cai, Sarah Caine, Leonardo Calò, Valeria Calvi, Mauricio Camarillo Sánchez, Rui Candeias, Vincenzo Capuano, Alessandro Capucci, Ronald Caputo, Tatiana Cárdenas Rizo, Francisco Cardona, Francisco Carlos da Costa Darrieux, Yan Carlos Duarte Vera, Antonio Carolei, Susana Carreño, Paula Carvalho, Susanna Cary, Gavino Casu, Claudio Cavallini, Guillaume Cayla, Aldo Celentano, Tae-Joon Cha, Kwang Soo Cha, Jei Keon Chae, Kathrine Chalamidas, Krishnan Challappa, Sunil Prakash Chand, Harinath Chandrashekar, Ludovic Chartier, Kausik Chatterjee, Carlos Antero Chavez Ayala, Aamir Cheema, Amjad Cheema, Lin Chen, Shih-Ann Chen, Jyh Hong Chen, Fu-Tien Chiang, Francesco Chiarella, Lin Chih-Chan, Yong Keun Cho, Jong-Il Choi, Dong Ju Choi, Guy Chouinard, Danny Hoi-Fan Chow, Dimitrios Chrysos, Galina Chumakova, Eduardo Julián José Roberto Chuquiure Valenzuela, Nicoleta Cindea Nica, David J. Cislowski, Anthony Clay, Piers Clifford, Andrew Cohen, Michael Cohen, Serge Cohen, Furio Colivicchi, Ronan Collins, Paolo Colonna, Steve Compton, Derek Connolly, Alberto Conti, Gabriel Contreras Buenostro, Gregg Coodley, Martin Cooper, Julian Coronel, Giovanni Corso, Juan Cosín Sales, Yves Cottin, John Covalesky, Aurel Cracan, Filippo Crea, Peter Crean, James Crenshaw, Tina Cullen, Harald Darius, Patrick Dary, Olivier Dascotte, Ira Dauber, Vicente Davalos, Ruth Davies, Gershan Davis, Jean-Marc Davy, Mark Dayer, Marzia De Biasio, Silvana De Bonis, Raffaele De Caterina, Teresiano De Franceschi, J. R. de Groot, José De Horta, Axel De La Briolle, Gilberto de la Pena Topete, Angelo Amato Vicenzo de Paola, Weimar de Souza, A. de Veer, Luc De Wolf, Eric Decoulx, Sasalu Deepak, Pascal Defaye, Freddy Del-Carpio Munoz, Diana Delic Brkljacic, N. Joseph Deumite, Silvia Di Legge, Igor Diemberger, Denise Dietz, Pedro Dionísio, Qiang Dong, Fabio Rossi dos Santos, Elena Dotcheva, Rami Doukky, Anthony D’Souza, Simon Dubrey, Xavier Ducrocq, Dmitry Dupljakov, Mauricio Duque, Dipankar Dutta, Nathalie Duvilla, A. Duygun, Rainer Dziewas Charles B. Eaton, William Eaves, L. A. Ebels-Tuinbeek, Clifford Ehrlich, Sabine Eichinger-Hasenauer, Steven J. Eisenberg, Adnan El Jabali, Mahfouz El Shahawy, Mauro Esteves Hernandes, Ana Etxeberria Izal, Rudolph Evonich, Oksana Evseeva, Andrey Ezhov, Raed Fahmy, Quan Fang, Ramin Farsad, Laurent Fauchier, Stefano Favale, Maxime Fayard, Jose Luis Fedele, Francesco Fedele, Olga Fedorishina, Steven R. Fera, Luis Gustavo Gomes Ferreira, Jorge Ferreira, Claudio Ferri, Anna Ferrier, Hugo Ferro, Alexandra Finsen, Brian First, Stuart Fischer, Catarina Fonseca, Luísa Fonseca Almeida, Steven Forman, Brad Frandsen, William French, Keith Friedman, Athena Friese, Ana Gabriela Fruntelata, Shigeru Fujii, Stefano Fumagalli, Marta Fundamenski, Yutaka Furukawa, Matthias Gabelmann, Nashwa Gabra, Niels Gadsbøll, Michel Galinier, Anders Gammelgaard, Priya Ganeshkumar, Christopher Gans, Antonio Garcia Quintana, Olivier Gartenlaub, Achille Gaspardone, Conrad Genz, Frédéric Georger, Jean-Louis Georges, Steven Georgeson, Evaldas Giedrimas, Mariusz Gierba, Ignacio Gil Ortega, Eve Gillespie, Alberto Giniger, Michael C. Giudici, Alexandros Gkotsis, Taya V. Glotzer, Joachim Gmehling, Jacek Gniot, Peter Goethals, Seth Goldbarg, Ronald Goldberg, Britta Goldmann, Sergey Golitsyn, Silvia Gómez, Juan Gomez Mesa, Vicente Bertomeu Gonzalez, Jesus Antonio Gonzalez Hermosillo, Víctor Manuel González López, Hervé Gorka, Charles Gornick, Diana Gorog, Venkat Gottipaty, Pascal Goube, Ioannis Goudevenos, Brett Graham, G. Stephen Greer, Uwe Gremmler, Paul G. Grena, Martin Grond, Edoardo Gronda, Gerian Grönefeld, Xiang Gu, Ivett Guadalupe Torres Torres, Gabriele Guardigli, Carolina Guevara, Alexandre Guignier, Michele Gulizia, Michael Gumbley, Albrecht Günther, Andrew Ha, Georgios Hahalis, Joseph Hakas, Christian Hall, Bing Han, Seongwook Han, Joe Hargrove, David Hargroves, Kenneth B. Harris, Tetsuya Haruna, Emil Hayek, Jeff Healey, Steven Hearne, Michael Heffernan, Geir Heggelund, J. A. Heijmeriks, Maarten Hemels, I. Hendriks, Sam Henein, Sung-Ho Her, Paul Hermany, Jorge Eduardo Hernández Del Río, Yorihiko Higashino, Michael Hill, Tetsuo Hisadome, Eiji Hishida, Etienne Hoffer, Matthew Hoghton, Kui Hong, Suk keun Hong, Stevie Horbach, Masataka Horiuchi, Yinglong Hou, Jeff Hsing, Chi-Hung Huang, David Huckins, kathy Hughes, A. Huizinga, E. L. Hulsman Kuo-Chun Hung, Gyo-Seung Hwang, Margaret Ikpoh, Davide Imberti, Hüseyin Ince, Ciro Indolfi, Shujiro Inoue, Didier Irles, Harukazu Iseki, C. Noah Israel, Bruce Iteld, Venkat Iyer, Ewart Jackson-Voyzey, Naseem Jaffrani, Frank Jäger, Martin James, Sung-Won Jang, Nicolas Jaramillo, Nabil Jarmukli, Robert J. Jeanfreau, Ronald D. Jenkins, Carlos Jerjes Sánchez, Javier Jimenez, Robert Jobe, Tomas Joen-Jakobsen, Nicholas Jones, Jose Carlos Moura Jorge, Bernard Jouve, Byung Chun Jung, Kyung Tae Jung, Werner Jung, Mikhail Kachkovskiy, Krystallenia Kafkala, Larisa Kalinina, Bernd Kallmünzer, Farzan Kamali, Takehiro Kamo, Priit Kampus, Hisham Kashou, Andreas Kastrup, Apostolos Katsivas, Elizabeth Kaufman, Kazuya Kawai, Kenji Kawajiri, John F. Kazmierski, P. Keeling, José Francisco Kerr Saraiva, Galina Ketova, AJIT Singh Khaira, Aleksey Khripun, Doo-Il Kim, Young Hoon Kim, Nam Ho Kim, Dae Kyeong Kim, Jeong Su Kim, June Soo Kim, Ki Seok Kim, Jin bae Kim, Elena Kinova, Alexander Klein, James J. Kmetzo, G. Larsen Kneller, Aleksandar Knezevic, Su Mei Angela Koh, Shunichi Koide, Anastasios Kollias, J. A. Kooistra, Jay Koons, Martin Koschutnik, William J. Kostis, Dragan Kovacic, Jacek Kowalczyk, Natalya Koziolova, Peter Kraft, Johannes A. Kragten, Mori Krantz, Lars Krause, B. J. Krenning, F. Krikke, Z. Kromhout, Waldemar Krysiak, Priya Kumar, Thomas Kümler, Malte Kuniss, Jen-Yuan Kuo, Achim Küppers, Karla Kurrelmeyer, Choong Hwan Kwak, Bénédicte Laboulle, Arthur Labovitz, Wen Ter Lai, Andy Lam, Yat Yin Lam, Fernando Lanas Zanetti, Charles Landau, Giancarlo Landini, Estêvão Lanna Figueiredo, Torben Larsen, Karine Lavandier, Jessica LeBlanc, Moon Hyoung Lee, Chang-Hoon Lee, John Lehman, Ana Leitão, Nicolas Lellouche, Malgorzata Lelonek, Radoslaw Lenarczyk, T. Lenderink, Salvador León González, Peter Leong-Sit, Matthias Leschke, Nicolas Ley, Zhanquan Li, Xiaodong Li, Weihua Li, Xiaoming Li, Christhoh Lichy, Ira Lieber, Ramon Horacio Limon Rodriguez, Hailong Lin, Gregory Y. H. Lip, Feng Liu, Hengliang Liu, Guillermo Llamas Esperon, Nassip Llerena Navarro, Eric Lo, Sergiy Lokshyn, Amador López, José Luís López-Sendón, Adalberto Menezes Lorga Filho, Richard S. Lorraine, Carlos Alberto Luengas, Robert Luke, Ming Luo, Steven Lupovitch, Philippe Lyrer, Changsheng Ma, Genshan Ma, Irene Madariaga, Koji Maeno, Dominique Magnin, Gustavo Maid, Sumeet K. Mainigi, Konstantinos Makaritsis, Rohit Malhotra, Rickey Manning, Athanasios Manolis, Helard Andres Manrique Hurtado, Ioannis Mantas, Fernando Manzur Jattin, Vicky Maqueda, Niccolo Marchionni, Francisco Marin Ortuno, Antonio Martín Santana, Jorge Martinez, Petra Maskova, Norberto Matadamas Hernandez, Katsuhiro Matsuda, Tillmann Maurer, Ciro Mauro, Erik May, Nolan Mayer, John McClure, Terry McCormack, William McGarity, Hugh McIntyre, Brent McLaurin, Feliz Alvaro Medina Palomino, Francesco Melandri, Hiroshi Meno, Dhananjai Menzies, Marco Mercader, Christian Meyer, Beat J. Meyer, Jacek Miarka, Frank Mibach, Dominik Michalski, Patrik Michel, Rami Mihail Chreih, Ghiath Mikdadi, Milan Mikus, Davor Milicic, Constantin Militaru, Sedi Minaie, Bogdan Minescu, Iveta Mintale, Tristan Mirault, Michael J. Mirro, Dinesh Mistry, Nicoleta Violeta Miu, Naomasa Miyamoto, Tiziano Moccetti, Akber Mohammed, Azlisham Mohd Nor, Michael Mollerus, Giulio Molon, Sergio Mondillo, Patrícia Moniz, Lluis Mont, Vicente Montagud, Oscar Montaña, Cristina Monti, Luciano Moretti, Kiyoo Mori, Andrew Moriarty, Jacek Morka, Luigi Moschini, Nikitas Moschos, Andreas Mügge, Thomas J. Mulhearn, Carmen Muresan, Michela Muriago, Wlodzimierz Musial, Carl W. Musser, Francesco Musumeci, Thuraia Nageh, Hidemitsu Nakagawa, Yuichiro Nakamura, Toru Nakayama, Gi-Byoung Nam, Michele Nanna, Indira Natarajan, Hemal M. Nayak, Stefan Naydenov, Jurica Nazlić, Alexandru Cristian Nechita, Libor Nechvatal, Sandra Adela Negron, James Neiman, Fernando Carvalho Neuenschwander, David Neves, Anna Neykova, Ricardo Nicolás Miguel, George Nijmeh, Alexey Nizov, Rodrigo Noronha Campos, Janko Nossan, Tatiana Novikova, Ewa Nowalany-Kozielska, Emmanuel Nsah, Juan Carlos Nunez Fragoso, Svetlana Nurgalieva, Dieter Nuyens, Ole Nyvad, Manuel Odin de Los Rios Ibarra, Philip O’Donnell, Martin O’Donnell, Seil Oh, Yong Seog Oh, Dongjin Oh, Gilles O’Hara, Kostas Oikonomou, Claudia Olivares, Richard Oliver, Rafael Olvera Ruiz, Christoforos Olympios, Anna omaszuk-Kazberuk, Joaquín Osca Asensi, eena Padayattil jose, Francisco Gerardo Padilla Padilla, Victoria Padilla Rios, Giuseppe Pajes, A. Shekhar Pandey, Gaetano Paparella, F. Paris, Hyung Wook Park, Jong Sung Park, Fragkiskos Parthenakis, Enrico Passamonti, Rajesh J. Patel, Jaydutt Patel, Mehool Patel, Janice Patrick, Ricardo Pavón Jimenez, Analía Paz, Vittorio Pengo, William Pentz, Beatriz Pérez, Alma Minerva Pérez Ríos, Alejandro Pérez-Cabezas, Richard Perlman, Viktor Persic, Francesco Perticone, Terri K. Peters, Sanjiv Petkar, Luis Felipe Pezo, Christian Pflücke, David N. Pham, Roland T. Phillips, Stephen Phlaum, Denis Pieters, Julien Pineau, Arnold Pinter, Fausto Pinto, R. Pisters, Nediljko Pivac, Darko Pocanic, Cristian Podoleanu, Alessandro Politano, Zdravka Poljakovic, Stewart Pollock, Jose Polo Garcéa, Holger Poppert, Maurizio Porcu, Antonio Pose Reino, Neeraj Prasad, Dalton Bertolim Précoma, Alessandro Prelle, John Prodafikas, Konstantin Protasov, Maurice Pye, Zhaohui Qiu, Jean-Michel Quedillac, Dimitar Raev, Carlos Antonio Raffo Grado, Sidiqullah Rahimi, Arturo Raisaro, Bhola Rama, Ricardo Ramos, Maria Ranieri, Nuno Raposo, Eric Rashba, Ursula Rauch-Kroehnert, Ramakota Reddy, Giulia Renda, Shabbir Reza, Luigi Ria, Dimitrios Richter, Hans Rickli, Werner Rieker, Tomas Ripolil Vera, Luiz Eduardo Ritt, Douglas Roberts, Ignacio Rodriguez Briones, Aldo Edwin Rodriguez Escudero, Carlos Rodríguez Pascual, Mark Roman, Francesco Romeo, E. Ronner, Jean-Francois Roux, Nadezda Rozkova, Miroslav Rubacek, Frank Rubalcava, Andrea M. Russo, Matthieu Pierre Rutgers, Karin Rybak, Samir Said, Tamotsu Sakamoto, Abraham Salacata, Adrien Salem, Rafael Salguero Bodes, Marco A. Saltzman, Alessandro Salvioni, Gregorio Sanchez Vallejo, Marcelo Sanmartín Fernández, Wladmir Faustino Saporito, Kesari Sarikonda, Taishi Sasaoka, Hamdi Sati, Irina Savelieva, Pierre-Jean Scala, Peter Schellinger, Carlos Scherr, Lisa Schmitz, Karl-Heinz Schmitz, Bettina Schmitz, Teresa Schnabel, Steffen Schnupp, Peter Schoeniger, Norbert Schön, Peter Schwimmbeck, Clare Seamark, Greg Searles, Karl-Heinz Seidl, Barry Seidman, Jaroslaw Sek, Lakshmanan Sekaran, Carlo Serrati, Neerav Shah, Vinay Shah, Anil Shah, Shujahat Shah, Vijay Kumar Sharma, Louise Shaw, Khalid H. Sheikh, Naruhito Shimizu, Hideki Shimomura, Dong-Gu Shin, Eun-Seok Shin, Junya Shite, Gerolamo Sibilio, Frank Silver, Iveta Sime, Tim A. Simmers, Narendra Singh, Peter Siostrzonek, Didier Smadja, David W. Smith, Marcelo Snitman, Dario Sobral Filho, Hassan Soda, Carl Sofley, Adam Sokal, Yannie Soo Oi Yan, Rodolfo Sotolongo, Olga Ferreira de Souza, Jon Arne Sparby, Jindrich Spinar, David Sprigings, Alex C. Spyropoulos, Dimitrios Stakos, Clemens Steinwender, Georgios Stergiou, Ian Stiell, Marcus Stoddard, Anastas Stoikov, Witold Streb, Ioannis Styliadis, Guohai Su, Xi Su, Wanda Sudnik, Kai Sukles, Xiaofei Sun, H. Swart, Janko Szavits-Nossan, Jens Taggeselle, Yuichiro Takagi, Amrit Pal Singh Takhar, Angelika Tamm, Katsumi Tanaka, Tanyanan Tanawuttiwat, Sherman Tang, Aylmer Tang, Giovanni Tarsi, Tiziana Tassinari, Ashis Tayal, Muzahir Tayebjee, J. M. ten Berg, Dan Tesloianu, Salem H. K. The, Dierk Thomas, Serge Timsit, Tetsuya Tobaru, Andrzej R. Tomasik., Mikhail Torosoff, Emmanuel Touze, Elina Trendafilova, W. Kevin Tsai, Hung Fat Tse, Hiroshi Tsutsui, Tian Ming Tu, Ype Tuininga, Minang Turakhia, Samir Turk, Wayne Turner, Arnljot Tveit, Richard Tytus, C. Valadão, P. F. M. M. van Bergen, Philippe van de Borne, B. J. van den Berg, C. van der Zwaan, M. Van Eck, Peter Vanacker, Dimo Vasilev, Vasileios Vasilikos, Maxim Vasilyev, Srikar Veerareddy, Mario Vega Miño, Asok Venkataraman, Paolo Verdecchia, Francesco Versaci, Ernst Günter Vester, Hubert Vial, Jason Victory, Alejandro Villamil, Marc Vincent, Anthony Vlastaris, Jürgen vom Dahl, Kishor Vora, Robert B. Vranian, Paul Wakefield, Ningfu Wang, Mingsheng Wang, Xinhua Wang, Feng Wang, Tian Wang, Alberta L. Warner, Kouki Watanabe, Jeanne Wei, Christian Weimar, Stanislav Weiner, Renate Weinrich, Ming-Shien Wen, Marcus Wiemer, Preben Wiggers, Andreas Wilke, David Williams, Marcus L. Williams, Bernhard Witzenbichler, Brian Wong, Ka Sing Lawrence Wong, Beata Wozakowska-Kaplon, Shulin Wu, Richard C. Wu, Silke Wunderlich, Nell Wyatt, John Wylie, Yong Xu, Xiangdong Xu, Hiroki Yamanoue, Takeshi Yamashita, Ping Yen Bryan Yan, Tianlun Yang, Jing Yao, Kuo-Ho Yeh, Wei Hsian Yin, Yoto Yotov, Ralf Zahn, Stuart Zarich, Sergei Zenin, Elisabeth Louise Zeuthen, Huanyi Zhang, Donghui Zhang, Xingwei Zhang, Ping Zhang, Jun Zhang, Shui Ping Zhao, Yujie Zhao, Zhichen Zhao, Yang Zheng, Jing Zhou, Sergio Zimmermann, Andrea Zini, Steven Zizzo, Wenxia Zong, L. Steven Zukerman

**Affiliations:** 1 Liverpool Centre for Cardiovascular Science, University of Liverpool, Liverpool John Moores University and Liverpool Heart & Chest Hospital, Liverpool, UK; 2 Department of Translational and Precision Medicine, Sapienza - University of Rome, Rome, Italy; 3 Division of Subacute Care, IRCCS Istituti Clinici Scientifici Maugeri, Milan, Italy; 4 Department of Clinical Sciences and Community Health, University of Milan, Milan, Italy; 5 Department of Clinical, Internal, Anesthesiological and Cardiovascular Sciences, Sapienza University of Rome, Rome, Italy; 6 Department of Thrombosis and Hemostasis, Leiden University Medical Center, Leiden, The Netherlands; 7 Department of Clinical Medicine, Aalborg Thrombosis Research Unit, Aalborg University, Aalborg, Denmark

**Keywords:** Atrial fibrillation, Risk stratification, Myocardial infarction, Cardiovascular mortality

## Abstract

The 2MACE score was specifically developed as a risk-stratification tool in atrial fibrillation (AF) to predict cardiovascular outcomes. We evaluated the predictive ability of the 2MACE score in the GLORIA-AF registry. All eligible patients from phase II/III of the prospective global GLORIA-AF registry were included. Major adverse cardiac events (MACEs) were defined as the composite outcome of stroke, myocardial infarction and cardiovascular death. Cox proportional hazards were used to examine the relationship between the 2MACE score and study outcomes. Predictive capability of the 2MACE score was investigated using receiver-operating characteristic curves. A total of 25,696 patients were included (mean age 71 years, female 44.9%). Over 3 years, 1583 MACEs were recorded. Patients who had MACE were older, with more cardiovascular risk factors and were less likely to be managed using a rhythm-control strategy. The median 2MACE score in the MACE and non-MACE groups were 2 (IQR 1–3) and 1 (IQR 0–2), respectively (p < 0.001). The 2MACE score was positively associated with an increase in the risk of MACE, with a score of ≥ 2 providing the best combination of sensitivity (69.6%) and specificity (51.6%), HR 2.47 (95% CI, 2.21–2.77). The 2MACE score had modest predictive performance for MACE in patients with AF (AUC 0.655 (95% CI, 0.641–0.669)). Our analysis in this prospective global registry demonstrates that the 2MACE score can adequately predict the risk of MACE (defined as myocardial infarction, CV death and stroke) in patients with AF. Clinical trial registration:http://www.clinicaltrials.gov. Unique identifiers: NCT01468701, NCT01671007 and NCT01937377

**Supplementary Information:**

The online version contains supplementary material available at 10.1007/s11239-023-02866-y.

## Highlights

- Patients with atrial fibrillation are at high risk for major adverse cardiac events.
- The 2MACE score can adequately predict the risk of major adverse cardiac events in patients with atrial fibrillation.
- These high-risk patients may benefit from intense and integrated care management strategies

## Introduction

Atrial fibrillation (AF) is substantially burdened with a high risk of mortality and cardiovascular events [1]. These have been long established, with frameworks such as the CHA_2_DS_2−_VASc score and ABC pathway in place for stroke risk stratification and AF management, respectively [1, 2]. More recent evidence suggests that AF is independently associated with a 2-fold risk of major adverse cardiovascular events (MACE) such as myocardial infarction (MI) and revascularisation procedures such as percutaneous coronary intervention (PCI) and coronary artery bypass graft (CABG) surgery, even in those without a prior history of coronary artery disease (CAD) [3–5]. The risk of MI increases by 47% in the presence of AF, with this excess risk rising to 71% in those with no previous cardiac history [6]. Of note, risk of MACE in patients with AF has been shown to be lower in those who are appropriately anticoagulated [7, 8]. The extent of this issue has inadvertently been met with a focus on establishing a reliable method for stratifying the risk of MACE, to identify high-risk subsets of AF patients.

A previous study by Pastori et al. led to the derivation of the 2MACE score for use in patients with AF, based on clinical predictors associated with MACEs such as fatal/non-fatal MI, cardiac revascularisation and cardiac mortality [9]. The 2MACE score comprises 2 points each for metabolic syndrome and age equal to or greater than 75, and 1 point each for MI/revascularisation, congestive heart failure (ejection fraction ≤ 40%), thromboembolism (stroke/transient ischemic attack [TIA]), thus ranging from 0 to 7. A score of 3 or more is considered high-risk, corresponding with a nearly 4-fold increase in the risk of MACE [9].

To date, the 2MACE score has only been validated in a few cohorts, largely retrospective with small number of patients, and in limited settings [10]. Therefore, we aimed to evaluate its predictive ability in a large, global contemporary prospective cohort of newly diagnosed patients with AF from the GLORIA-AF (Global Registry on Long-Term Oral Anti-thrombotic Treatment In Patients With Atrial Fibrillation) registry.

## Methods

### Study design and population

GLORIA-AF is a prospective, observational, global registry programme of patients who were recruited from 935 participating centres across 38 countries in Asia, Europe, North America, Latin America, and Africa/Middle East. The study design has previously been described [11]. Briefly, consecutive adults with newly diagnosed AF (< 3 months before baseline visit) and an increased risk of stroke (CHA_2_DS_2_-VASc ≥ 1) were enrolled. This study focused on patients from GLORIA-AF phase II and III. These patients were enrolled between 2011 and 2020. Eligible patients with follow-up data in relation to MACE were included. Main exclusion criteria were presence of mechanical heart valve or valvular disease necessitating valve replacement, previous oral anticoagulation with vitamin K oral antagonist over 60 days, a reversible cause of AF, indication for anticoagulation other than AF, and life expectancy of less than 1 year. Ethics approval was obtained from local institutional review boards, informed consent was obtained from patients, and the study was performed in accordance with the Declaration of Helsinki.

### Data collection and definition

Data on demographics, comorbidities and therapies were collected at enrolment with standardised, prospectively designed data collection tools. Creatinine clearance (CrCl) was assessed using the Cockcroft-Gault equation [12]. AF classification was determined according to the European Society of Cardiology recommendations [1]. Severity of AF-related symptoms was ascertained using the European Heart Rhythm Association classification [13]. CHADS_2_, CHA_2_DS_2_-VASc and HAS-BLED scores were calculated as previously described [14–16]. The 2MACE score was determined by assigning 2 points for metabolic syndrome and age ≥ 75 years, and 1 point for previous MI or CABG, congestive heart failure and prior thromboembolism [10]. Metabolic syndrome was defined according to the World Health Organisation criteria as the presence of diabetes mellitus and 2 other risk factors including hypertension, hypercholesterolaemia or BMI over 30 kg/m^2^ [17].

### Study outcomes and follow-up

Outcomes of interest were MACEs, defined as the composite outcome of pre-specified events including cardiovascular (CV) death, MI and stroke, and its individual components. MI was defined as the development of significant Q-waves in at least 2 adjacent electrocardiogram leads, or at least 2 of the following 3 criteria: (i) typical prolonged severe chest pain of at least 30 min; (ii) electrocardiographic changes suggestive of MI including ST-changes or T wave inversion in the electrocardiogram; (iii) elevation of troponin or creatinine kinase-MB to more than upper level of normal or, if creatinine kinase-MB was elevated at baseline, re-elevation to more than 50% increase above the previous level. Stroke was defined as an acute onset of a focal neurological deficit of presumed vascular origin lasting for 24 h or more, or that resulting in death. In phase II, follow-up for the dabigatran cohort was for 2 years, with scheduled visits at 3, 6, 12, and 24 months. In phase III, follow-up for all patients was conducted for 3 years, with scheduled visits at 6, 12, 24, and 36 months. CV death was defined as death due to stroke, non-central nervous system arterial embolism, pulmonary embolism, MI, haemorrhage, sudden cardiac death, pump failure, peripheral embolus or aortic dissection/rupture. Subgroup analyses were performed in patients equal or greater than 75 years old, female sex and patients with prior stroke.

### Statistical analysis

Continuous variables were presented as median and interquartile range (IQR) and assessed for differences with Kruskal-Wallis test. Categorical variables were presented as count and percentage and assessed for differences with chi-squared test. The incidence rates (number of events/person-years at risk) with 95% confidence intervals for the outcomes of interest were calculated using previously described methods [18]. The relationship between the 2MACE score and study outcomes were analysed using Cox proportional hazards analyses.

Potential confounders were accounted for using a multivariable model with pre-specified selection of covariates including sex, creatinine clearance, type of atrial fibrillation, left ventricular hypertrophy, prior bleeding, peripheral artery disease, chronic obstructive pulmonary disease, AF ablation, oral anticoagulation use, antiplatelet use, anti-arrhythmic drug therapy, angiotensin-converting enzyme inhibitor, angiotensin receptor blocker, beta-blocker, digoxin, statin and diuretic therapy.

Predictive capability of the 2MACE score for MACEs was investigated using receiver-operating characteristic curves, and the performance was tested against the CHA_2_DS_2_-VASc score using DeLong’s method [19]. Area under the curve (AUC) was used to reflect the c-index, which represents the ability of scores to predict events. A two-sided p value of less than 0.05 was considered statistically significant. Statistical analyses were performed using R 4.0.3 (Vienna, Austria).

## Results

A total of 25,696 patients with 11,527 (44.9%) females and a median age of 71 (IQR 64–78) years were included. The patient flow is shown in Supplementary Fig. 1.

### Baseline characteristics

Baseline characteristics based on patients which reported MACEs are described in Table 1. Patients who suffered from a MACE were older, more likely to be male, and had higher BMI, poorer renal function by CrCl and greater burden of comorbidities including hypertension, hypercholesterolaemia, diabetes mellitus, CAD, prior MI, heart failure, left ventricular hypertrophy, prior thromboembolism, prior bleeding, peripheral artery disease and chronic obstructive pulmonary disease. As a result, patients who had a MACE had higher risk of stroke by CHA_2_DS_2_-VASc score, and major bleeding by HAS-BLED score. At baseline, MACE patients had a median 2MACE score of 2 (IQR 1–3) compared to 1 (IQR 0–2) in those without a MACE. The distribution of patients according to the 2MACE score is presented in Supplementary Fig. 2.

**Table 1:** Baseline characteristics

Baseline characteristics	MACE (n = 1583)	No MACE (n = 24,113)	p value
Age (years), median (IQR)	76.0 (69.0–81.0)	71.0 (64.0–77.0)	< 0.001
Female sex, n (%)	654 (41.3%)	10,873 (45.1%)	0.004
Heart rate (bpm), median (IQR)	78 (67–90)	76 (65–90)	0.004
sBP (mmHg), median (IQR)	130 (120–143)	130 (120–142)	0.392
BMI (kg/m^2^), median (IQR)	26.7 (23.9–30.7)	27.6 (24.6–31.6)	< 0.001
CrCl (mL/min), median (IQR)	62.0 (44.1–83.3)	76.7 (58.4–99.6)	< 0.001
AF classification, n (%)			< 0.001
Paroxysmal	799 (50.5%)	13,495 (56.0%)	
Persistent	578 (36.5%)	8243 (34.2%)	
Permanent	206 (13.0%)	2375 (9.9%)	
EHRA classification, n (%)			< 0.001
I	584 (38.5%)	8073 (35.4%)	
II	469 (30.9%)	8677 (38.1%)	
III	365 (24.1%)	4670 (20.5%)	
IV	99 (6.5%)	1356 (6.0%)	
Comorbidities, n (%)			
Hypertension	1261 (79.8%)	18,034 (75.0%)	< 0.001
Hypercholesterolaemia	712 (46.2%)	9457 (40.3%)	< 0.001
Diabetes mellitus	494 (31.2%)	5423 (22.5%)	< 0.001
Coronary artery disease	517 (33.5%)	4279 (18.2%)	< 0.001
Prior myocardial infarction	333 (21.1%)	2088 (8.7%)	< 0.001
Congestive heart failure	564 (36.0%)	5014 (21.0%)	< 0.001
Left ventricular hypertrophy	349 (23.0%)	4362 (19.0%)	< 0.001
Prior thromboembolism	352 (22.2%)	3428 (14.2%)	< 0.001
Prior stroke	278 (17.6%)	2475 (10.3%)	< 0.001
Prior bleeding	116 (7.4%)	1234 (5.2%)	< 0.001
Peripheral artery disease	93 (5.9%)	631 (2.6%)	< 0.001
COPD	148 (9.4%)	1367 (5.7%)	< 0.001
2MACE score, median (IQR)	2 (1–3)	1 (0–2)	< 0.001
CHA_2_DS_2_-VASc score, median (IQR)	4 (3–5)	3 (2–4)	< 0.001
HAS-BLED score, median (IQR)	2 (1–2)	1 (1–2)	< 0.001

*AF* atrial fibrillation, *BMI* body mass index, *COPD* chronic obstructive pulmonary disease, *CrCl* creatinine clearance,  *EHRA* European Heart Rhythm Association, *IQR* interquartile range, *LVEF* left ventricular ejection fraction, *MACE* major adverse cardiovascular event, *sBP* systolic blood pressure

### Medication use and therapies

Those patients which reported a MACE were less likely to have received AF ablation and had lower uptake of anticoagulation and anti-arrhythmic drug compared to those which did not report the outcome. However, there was greater use of antiplatelet, angiotensin-converting enzyme inhibitor, beta-blocker, digoxin, diuretic and statin therapy among MACE patients (Supplementary Table 1).

### Relationship between 2MACE score and outcomes

During a median follow-up period of 3.0 (IQR 2.3–3.1) years, there were 1583 (6.2%) MACEs defined as a composite outcome of CV death, MI and stroke, 813 (3.2%) CV death, 432 (1.7%) MI and 681 (2.7%) stroke (Table 2). The incidence of MACEs per 100 person-years was 2.31 (95% CI, 2.20–2.43), of which CV death 1.17 (95% CI, 1.09–1.25), MI 0.62 (95% CI, 0.56–0.68) and stroke 0.98 (95% CI, 0.91–1.06).

**Table 2:** Association between 2MACE score and major adverse cardiovascular events

Outcomes	n (%)	Incidence per 100 PYs (95% CI)	Relationship with 2MACE score		
			Univariate HR (95% CI)	aHR* (95%)	p value
MACE	1583 (6.2%)	2.31 (2.20–2.43)	1.45 (1.40–1.50)	1.33 (1.27–1.38)	< 0.001
CV death or myocardial infarction	1102 (4.3%)	1.59 (1.50–1.69)	1.54 (1.48–1.60)	1.36 (1.30–1.43)	< 0.001
CV death	813 (3.2%)	1.17 (1.09–1.25)	1.59 (1.52–1.66)	1.39 (1.31–1.47)	< 0.001
Myocardial infarction	432 (1.7%)	0.62 (0.56–0.68)	1.46 (1.37–1.55)	1.32 (1.22–1.43)	< 0.001
Stroke	681 (2.7%)	0.98 (0.91–1.06)	1.29 (1.23–1.36)	1.24 (1.16–1.32)	< 0.001

*aHR* adjusted hazard ratio, *CI* confidence interval, *CV* cardiovascular, *HR* hazard ratio, *MACE* major adverse cardiovascular event, *PY* person-years

*Adjusted for gender, creatinine clearance, type of atrial fibrillation, left ventricular hypertrophy, prior bleeding, peripheral artery disease, chronic obstructive pulmonary disease, AF ablation, oral anticoagulation use, antiplatelet use, anti-arrhythmic drug therapy, angiotensin-converting enzyme inhibitor, angiotensin receptor blocker, beta-blocker, digoxin, statin and diuretic therapy

The 2MACE score was associated with a significant increase in risk of MACE (HR 1.45 [95% CI, 1.40–1.50]), as well as separately for CV death or MI (HR 1.54 [95% CI, 1.48–1.60]), CV death (HR 1.59 [95% CI, 1.52–1.66]), MI (HR 1.46 [95% CI, 1.37–1.55]) and stroke (HR 1.29 [95% CI, 1.23–1.36]). After adjustment for confounders, an increase in the 2MACE score remained associated to the occurrence of MACEs (aHR 1.33 [95% CI, 1.27–1.38]) and the single components of the composite outcome, namely CV death or MI (aHR 1.36 [95% CI, 1.30–1.43]), CV death (aHR 1.39 [95% CI, 1.31–1.47]), MI (aHR 1.32 [95% CI, 1.22–1.43]) and stroke (aHR 1.24 [95% CI, 1.16–1.32]).

### Predictive ability of 2MACE score

**Fig. 1:**
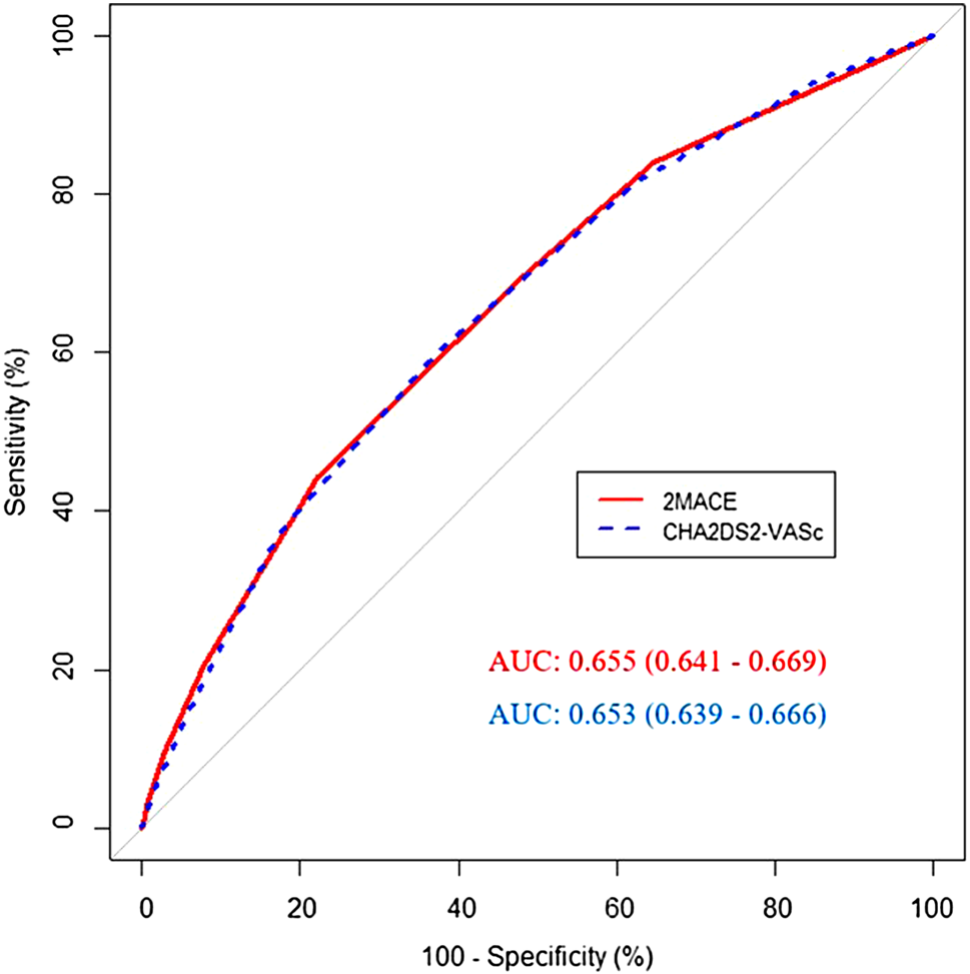
Receiver-operating characteristic curves comparison for major adverse cardiovascular events (MACE) with the 2MACE and CHA2DS2-VASc score

Using receiver-operating characteristic curve analysis, the AUC of the 2MACE score for prediction of MACE was 0.655 (95% CI, 0.641–0.669) (Fig. 1). There was no statistical difference between the predictive ability of the 2MACE score and CHA_2_DS_2_-VASc score (0.653 [95% CI, 0.639–0.666]) for MACE (p = 0.236). The sensitivity and specificity with corresponding HRs for MACEs occurrence at each point of the 2MACE score is presented in Table 3. A cut-off of equal or more than 2 points for the 2MACE score provided good sensitivity (69.6%) and acceptable specificity (51.6%) with a HR of 2.47 (95% CI, 2.21–2.77).

**Table 3:** Sensitivity, specificity and hazard ratios for MACE at different thresholds of the 2MACE score

2MACE score	Sensitivity (%)	Specificity (%)	HR (95% CI)	p value
≥ 1	84.1	35.4	2.89 (2.50–3.33)	< 0.001
≥ 2	69.6	51.6	2.47 (2.21–2.77)	< 0.001
≥ 3	44.3	77.8	2.81 (2.53–3.12)	< 0.001
≥ 4	20.7	92.0	2.97 (2.62–3.38)	< 0.001
≥ 5	9.2	97.2	3.49 (2.92–4.17)	< 0.001
≥ 6	2.6	99.3	4.12 (3.00–5.66)	< 0.001
≥ 7	0.3	99.9	4.81 (2.00–11.57)	< 0.001

*CI* confidence interval, *HR* hazard ratio, *MACE* major adverse cardiovascular event

### Subgroup analysis

**Fig. 2:**
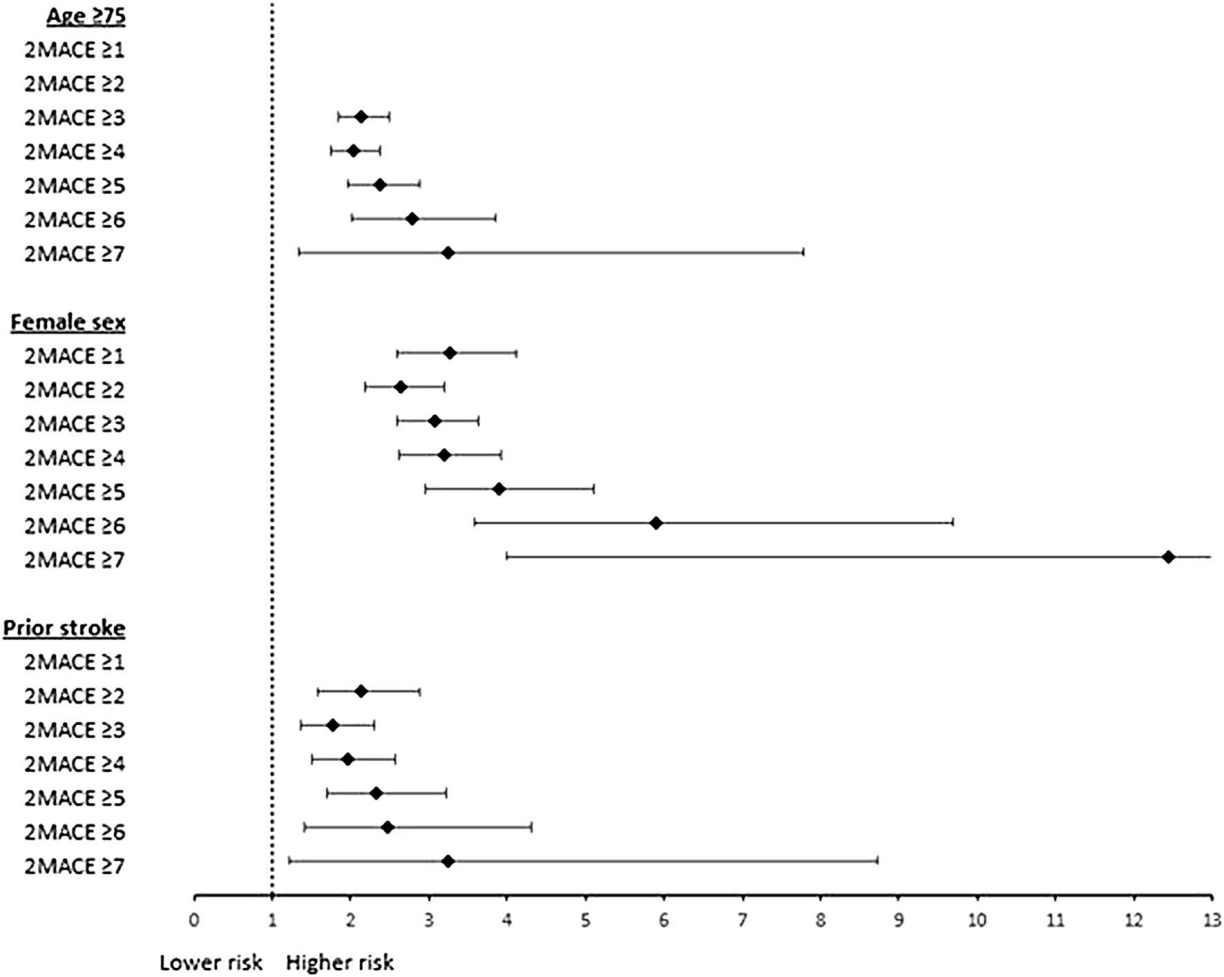
Results of the 2MACE score for prediction of MACE in subgroups of patients equal of greater than 75 years, female sex and patients with prior stroke

Results of the 2MACE score for prediction of MACEs in specific subgroups is shown in Fig. 2. Overall, the risk of MACEs increased with higher 2MACE score in each of the subgroups studied.

## Discussion

The principal findings of our study from a global contemporary cohort of anticoagulated patients with AF were as follows: (i) the 2MACE score was associated with a significantly increased risk of MACE, CV death, MI and stroke, (ii) the predictive ability of the 2MACE score for MACE including stroke was adequate and comparable, but not superior to that of the CHA_2_DS_2_-VASc score; and (iii) a 2MACE score of equal or greater than 2 may be useful for identifying high-risk subgroups.

To date, this is the largest study validating the predictive performance of the 2MACE score in a global prospective cohort of anticoagulated patients who were predominantly treated with non-vitamin K antagonist oral anticoagulants. Further, as this was based on a multi-centre registry, the results are more likely to be applicable to a wider range of patients from different geographical regions and ethnicities, as opposed to that from most existing studies which were based on single-centre cohorts, mostly on vitamin K antagonists.

In our analysis, 6.2% of the entire study population had a MACE, at an incidence rate of 2.31 events per 100 person-years. This was lower compared to the internal derivation cohort (10.9%) but similar to that of the external validation cohort (6.2%) in the study by Pastori et al. [9]. These differences may be attributable to the cohort sizes which were significantly smaller in contrast to the GLORIA-AF cohort, variations in the baseline characteristics and the differing follow-up durations. Nonetheless, it illustrates that AF patients remain at high residual risk of MACE despite being on oral anticoagulation [20–22]. This highlights the need for a more holistic or integrated care approach to AF management [23] and other chronic long term conditions [2, 24, 25]. In AF, such an approach has been associated with improved clinical outcomes [26, 27], leading to its incorporation into guidelines [28].

Our data demonstrated that an increase in the 2MACE score was positively associated with a graded increase in the risk of MACE. A 2MACE score of equal or greater than 2 had the best combination of sensitivity (69.6%) and specificity (51.6%) for MACE in our study, similar to the Murcia AF cohort [29]. Both sensitivity and specificity were inversely related, with sensitivity highest at 84.1% for a 2MACE score of equal or more than 1 (i.e. the probability for ruling out MACE was highest with lower scores), and specificity highest at 99.9% for a score equal or more than 7 (i.e. the probability of ruling out false positive results was highest with higher scores). In contrast, a 2MACE score of equal or more than 3 resulted in the best balance between the 2 in the validation studies by Pastori et al. (sensitivity 83%, specificity 68%) and Polovina et al. (sensitivity 74.6%, specificity 81.8%) [9, 30]. The corresponding sensitivity for each of the 2MACE scores were higher in their studies but these figures could represent spectrum effects associated with the clinical variability between study populations, which can influence performance of such tools [31].

Overall, we found that the 2MACE score had a moderate predictive ability for MACE in patients with AF. Although the c-index for predicting MACE was high at 0.79 (95% CI, 0.71–0.90) in the internal derivation cohort, this was 0.66 in the external validation cohort in the study by Pastori et al.[9]. Similarly, Rivera-Caravaca et al. derived c-indexes of 0.662 (95% CI, 0.625–0.697) and 0.656 (95% CI, 0.593–0.719) for discriminating MACE in the Murcia AF and FANTASIIA cohorts, on par with our results [29]. In contrast, the 2MACE score performed better in the analysis by Polovina et al. [30], with a marginally higher c-statistic of 0.699 (95% CI, 0.648–0.750). Notable differences between this study compared to the others included the younger population, lower CHA_2_DS_2_-VASc score, exclusion of patients with known CAD and the large proportion of patients who were managed with a rhythm control strategy, suggesting that this was lower risk population, and also raising the question whether the 2MACE score performs better in lower risk populations.

In addition to the above, the 2MACE score has been utilised in other real-world studies. In the ATHERO-AF study, the 2MACE score was utilised to categorise patients into low- and high-risk groups using a cut off of equal or more than 3 [32]. Further, it has been evaluated in AF cohorts with comorbidities such as chronic kidney disease and obstructive sleep apnoea to stratify CV risk with reasonable reliability [10, 33].

Over a median follow-up period of 3 years, a total of 1583 (6.2%) MACE were observed in the GLORIA-AF cohort, of which the majority were MI and CV death, with stroke being less frequent. Given this, it seems prudent to have a risk-prediction tool for CV events, the same way several exist for stroke risk-stratification. Nonetheless, we did not demonstrate a significant difference in the predictive ability of the 2MACE compared to CHA_2_DS_2_-VASc score for MACE in this cohort. As per previous studies, the 2MACE score was significantly associated with an increased risk of all outcomes with the strongest association with CV death.

The 2MACE score is a convenient tool that can be used with ease, as the variables it encompasses are clinical and readily available from patient histories. Our study validates the ability of this tool in predicting the risk of MACE in patients with AF. The potential for improving its predictive performance exists and studies focusing on this are already underway. In a recent study, incorporation of microRNAs which have been implicated in thromboembolic disease, in the 2MACE score significantly improved its predictive performance, increasing the c-statistic to 0.762 [29]. The biomarker-based risk scores are also non-specific and may be predictive of outcomes beyond what they were proposed for [34]. However, such biomarkers are not widely used and focus on clinical variables that are easily accessible may enhance clinical uptake and utility. The specific role of the 2MACE score in influencing treatment decisions has not been formally tested. Nonetheless, the score highlights high-risk patients who may benefit the most from optimisation of their cardiovascular risk profile. Ultimately, the benefits and risk of each treatment will need to be balanced at an individual level though we have shown that the 2MACE score can be used to provide vital information in terms of risk. More research is needed to evaluate the role of the 2MACE score to guide the management of patients with AF.

### Limitations

Although the GLORIA-AF registry was a prospective registry, our analysis was conducted retrospectively and should therefore be interpreted with caution. Moreover, there was possible misclassification and selection biases due to the observational study design. Data on medication compliance and control of CV risk factors which may have influenced outcomes was not available. Given that only patients with newly diagnosed AF were enrolled, the results presented here may not be applicable to the wider AF population. Though used for comparison in this study, the CHA_2_DS_2_-VASc score was not designed for the prediction of MACEs. Lastly, even though our results complement current evidence and provide useful insights, a direct comparison with other studies on this subject should be avoided due to differences in the definitions and study outcomes.

## Conclusion

The results of this large global prospective study indicate that the 2MACE score can adequately predict the risk of MACE such as MI, CV death as well as stroke in AF patients, in the short- and intermediate term, and supports its use as a risk-stratification tool for MACE in the AF cohort. It may be used to identify high-risk patients who will stand to benefit from more intense and integrated care measures in terms of lifestyle and risk factor modification as well as management strategies such as a rhythm control approach.

### Supplementary Information

Below is the link to the electronic supplementary material.

Supplementary material 1 (DOCX 267.6 kb)

## Data Availability

Data supporting this study are available through the data contributors Boehringer Ingelheim through Vivli, Inc.
