# Correction: Validating the predictive ability of the 2MACE score for major adverse cardiovascular events in patients with atrial fibrillation: results from phase II/III of the GLORIA-AF registry

**DOI:** 10.1007/s11239-023-02904-9

**Published:** 2023-10-09

**Authors:** Wern Yew Ding, Ameenathul Mazaya Fawzy, Giulio Francesco Romiti, Marco Proietti, Daniele Pastori, Menno V. Huisman, Gregory Y. H. Lip, Ameenathul Mazaya Fawzy, on behalf of the GLORIA-AF Investigators

**Affiliations:** 1 Liverpool Centre for Cardiovascular Science, University of Liverpool, Liverpool John Moores University and Liverpool Heart & Chest Hospital, Liverpool, UK; 2 Department of Translational and Precision Medicine, Sapienza - University of Rome, Rome, Italy; 3 Division of Subacute Care, IRCCS Istituti Clinici Scientifici Maugeri, Milan, Italy; 4 Department of Clinical Sciences and Community Health, University of Milan, Milan, Italy; 5 Department of Clinical, Internal, Anesthesiological and Cardiovascular Sciences, Sapienza University of Rome, Rome, Italy; 6 Department of Thrombosis and Hemostasis, Leiden University Medical Center, Leiden, The Netherlands; 7 Department of Clinical Medicine, Aalborg Thrombosis Research Unit, Aalborg University, Aalborg, Denmark

**Journal of Thrombosis and Thrombolysis**

10.1007/s11239-023-02866-y

In the original publication of the article the GLORIA-AF Investigators member “Anastasios Kollias” was incorrectly written as “Athanasios Kollias.” Now it has been corrected. The original article has been corrected.

