# Supplementary material for: Validating the predictive ability of the 2MACE score for major adverse cardiovascular events in patients with atrial fibrillation: results from phase II/III of the GLORIA-AF registry
Source: J Thromb Thrombolysis. 2023 Aug 11;57(1):39–49. doi: 10.1007/s11239-023-02866-y (PMC10830583; doi:10.1007/s11239-023-02866-y)
Supplement: Supplementary file 1 — Supplementary material 1 (DOCX 267.6 kb) [file 11239_2023_2866_MOESM1_ESM.docx]

Supplementary Materials

Validating the Predictive Ability of the 2MACE score for Major Adverse Cardiovascular Events in Patients with Atrial Fibrillation:

Results from Phase II/III of the GLORIA-AF Registry

Short title: **2MACE score in AF**

Wern Yew Ding^1^; Ameenathul Mazaya Fawzy^1^; Giulio Francesco Romiti^1,2^; Marco Proietti^1,3,4^, Daniele Pastori^1,5^, Menno V Huisman^6*^; Gregory Y. H. Lip^1,7*^; on behalf of the GLORIA-AF Investigators^8^

^1^ Liverpool Centre for Cardiovascular Science at University of Liverpool, Liverpool John Moores University and Liverpool Heart & Chest Hospital, Liverpool, United Kingdom; ^2^Department of Translational and Precision Medicine, Sapienza - University of Rome, Rome, Italy; ^3^Division of Subacute Care, IRCCS Istituti Clinici Scientifici Maugeri, Milan, Italy; ^4^Department of Clinical Sciences and Community Health, University of Milan, Milan, Italy; ^5^Department of Clinical, Internal, Anesthesiological and Cardiovascular Sciences, Sapienza University of Rome; ^6^Department of Thrombosis and Hemostasis, Leiden University Medical Center, Leiden, the Netherlands; ^7^Aalborg Thrombosis Research Unit, Department of Clinical Medicine, Aalborg University, Aalborg, Denmark; ^8^Listed in Appendix.

[*Drs Huisman and Lip and co-Chairs of the GLORIA-AF registry]

Corresponding author:

Prof Gregory Y H Lip [gregory.lip@liverpool.ac.uk](mailto:gregory.lip@liverpool.ac.uk)


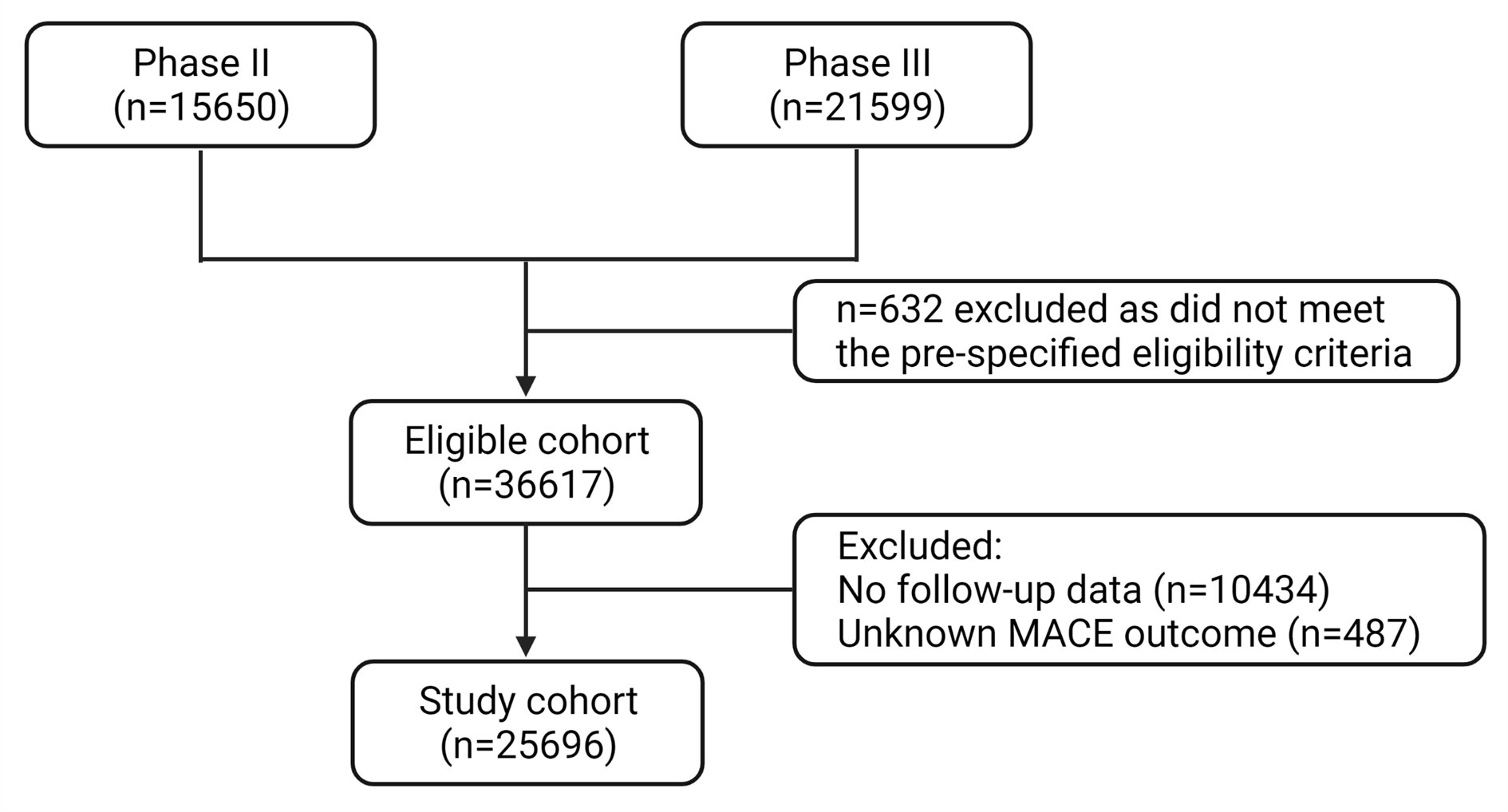


**Supplementary Figure 1**. Flow chart of patient selection.


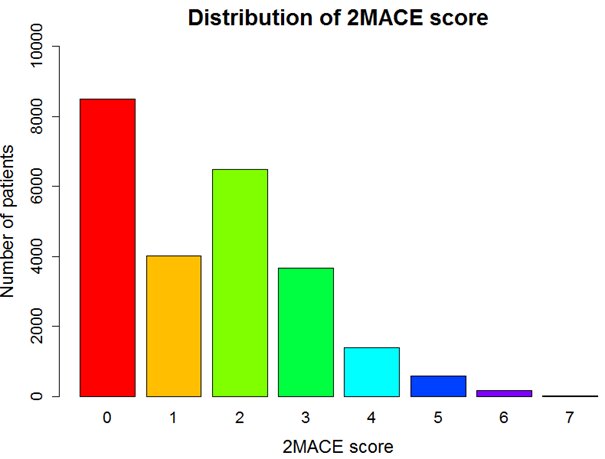


**Supplementary Figure 2**. Distribution of patients based on 2MACE score.

**Supplementary Table 1**. Medication use and therapies at enrolment

| **Medication use and therapies** | **MACE**  **(n=1583)** | **No MACE**  **(n=24113)** | **p value** |
| --- | --- | --- | --- |
| AF ablation | 14 (0.9%) | 429 (1.8%) | 0.010 |
| Anticoagulation, n (%) | 1266 (80.0%) | 20738 (86.0%) | <0.001 |
| Apixaban | 290 (18.3%) | 4124 (17.1%) |  |
| Dabigatran | 375 (23.7%) | 8225 (34.1%) |  |
| Edoxaban | 18 (1.1%) | 308 (1.3%) |  |
| Rivaroxaban | 236 (14.9%) | 3707 (15.4%) |  |
| VKA | 347 (21.9%) | 4374 (18.1%) |  |
| Antiplatelet, n (%) | 564 (35.6%) | 5620 (23.3%) | <0.001 |
| Anti-arrhythmic drug, n (%) | 344 (21.7%) | 6290 (26.1%) | <0.001 |
| ACE-i, n (%) | 526 (33.2%) | 7282 (30.2%) | 0.012 |
| Angiotensin receptor blocker, n (%) | 398 (25.1%) | 6196 (25.7%) | 0.646 |
| Beta-blocker, n (%) | 1043 (65.9%) | 15130 (62.7%) | 0.013 |
| Digoxin, n (%) | 182 (11.5%) | 1927 (8.0%) | <0.001 |
| Diuretic, n (%) | 753 (47.6%) | 8894 (36.9%) | <0.001 |
| Statin, n (%) | 849 (53.6%) | 10653 (44.2%) | <0.001 |

AF, atrial fibrillation; ACE-i, angiotensin-converting enzyme inhibitor; MACE, major adverse cardiovascular event; VKA, vitamin K antagonist.
